# Contraceptive use and maternal mortality in Indonesia: a community-level ecological analysis

**DOI:** 10.1186/s12978-020-01022-6

**Published:** 2021-02-17

**Authors:** Riznawaty Imma Aryanty, NohanArum Romadlona, Besral Besral, Elvi Debora P. Panggabean, Budi Utomo, Richard Makalew, Robert J. Magnani

**Affiliations:** 1 UNFPA Indonesia, Jakarta, Indonesia; 2 Department of Population and Biostatistics, Faculty of Public Health, University of Indonesia, Jakarta, Indonesia; 3 Faculty of Public Health, University of Indonesia, Jakarta, Indonesia

**Keywords:** Contraceptive use, Maternal mortality, Community level, Indonesia

## Abstract

**Background:**

Prior studies have shown that contraceptive use reduces maternal mortality independently of other maternal health services. The present study took advantage of geographically detailed Indonesian data to study the interplay between contraceptive use and other risk and protective factors for maternal mortality at the community level, a level of analysis where the protective effects of family planning can be best understood.

**Methods:**

Data from the 2015 Intercensal Population Survey (SUPAS) and the 2014 Village Potential Survey (PODES) were used to construct a series of census block-level variables measuring key risk and protective factors for maternal mortality. The relationships between these factors and maternal mortality, measured via natural log-transformation of past five-year maternal mortality ratios in each of the 40,748 census blocks were assessed via log-linear regressions.

**Results:**

Higher community maternal mortality ratios were associated with lower community contraceptive prevalence, higher percentage of parity four-plus births, higher proportion of poor households, lower population density of hospitals, higher density of traditional birth attendants (TBA), and residence outside of Java-Bali. For every percentage point increase in CPR, community maternal mortality ratios were lower by 7.0 points (95% CI = 0.9, 14.3). Community-level household wealth was the strongest predictor of maternal mortality.

**Conclusions:**

Community contraceptive prevalence made a significant contribution to reducing maternal mortality net of other risk and protective factors during 2010–2015. Increased health system responsiveness to the needs of pregnant women and reductions in socioeconomic and geographic disparities in maternal health services will be needed for Indonesia to reach the 2030 SDG maternal mortality goal.

## Background

Maternal mortality is unacceptably high in many parts of the world, including in Indonesia [1]. The latest Government of Indonesia estimate indicates a maternal mortality ratio (MMR) of 305 per 100,000 live births in 2015 [2]. Although there is considerable variability in the available estimates of the MMR in Indonesia, all available estimates indicate that maternal mortality is higher than it should be given the country’s level of gross national income (GNI) and health system development. High maternal mortality reflects weaknesses in health service delivery systems and often indicates inequitable access to health services, with the poor and adolescents being among the most vulnerable [3].

Family planning is one of core set of interventions that have been shown to reduce maternal mortality, the others being (1) skilled care during pregnancy and childbirth; (2) timely emergency obstetric care; and (3) immediate postnatal care [3]. While the latter three interventions focus on reducing risk among women who are or were recently pregnant, family planning lowers maternal mortality risk by reducing (1) the number of pregnancies that occur and (2) the proportion of pregnancies that are deemed to be “higher-risk” [4–6]. Fewer pregnancies translate into a reduction in the number of times that women are exposed to the risk of maternity-related mortality, an impact that compounds over time as fewer births yields successive smaller cohorts of women of reproductive age. Contraceptive use is a key direct determinant of fertility reduction [5, 7, 8], the other “proximate determinants” being marriage/sexual exposure, postpartum infecundability and induced abortion [7]. Contraceptive use also lowers the risk of maternal mortality per birth, as measured by the MMR, by preventing high-risk births; that is, births to women at the extremes of maternal age and parity and short-interval births [5, 9, 10]. Family planning has been estimated to have reduced maternal mortality levels in various countries by 6% to 60% [11, 12] – 44% globally [6], as well as lowering infant mortality and abortion rates, especially unsafe abortions [13, 14]. Mbizvo and Burke [15] estimate that globally family planning could prevent up to 30% of maternal deaths going forward.

Most prior analyses of the impact of family planning on maternal mortality have been undertaken using national or cross-national data. The present article sought to assess the contributions of family planning to reduced maternal mortality at the community level, a level of analysis where the interplay between contraceptive use and other maternal mortality risk and protective factors can be best understood. Because maternal deaths are a relatively infrequent event even in countries with high levels of maternal mortality, the data needed to undertake community-level analyses are rarely available. The present study took advantage of two sources of data—the 2015 Indonesian Intercensal Population Survey (SUPAS) [2] and the 2014 Village Potential Census (PODES) [16]—that are well-suited to support community-level analyses to quantify the effects of contraceptive use on maternal mortality net of the effects of a wide variety of other key determinants, including the local supply environment for maternal health services.

## Methods

### Data sources

The first primary data source, the Intercensal Population Survey (SUPAS), is conducted every 10 years at the mid-point between decennial population censuses [2]. The 2015 SUPAS collected a wealth of household and individual data relevant to the present article. Sample respondents were chosen using a stratified, two-stage cluster sampling scheme. The primary sampling unit was the census block, a geographically defined unit containing 80–120 households. A total of 40,728 census blocks were randomly chosen at the first stage of sample selection with probability proportional to estimated size (PPES) and allocated to provinces proportionally to provincial population size. As one priority of the 2015 SUPAS was to collect data on maternal mortality, a special sampling scheme was used to select households at the second stage of sample selection. In each selected census block, a sample of 16 households was chosen by first selecting with certainty households that reported maternal deaths in the previous five years (maximum of 8 households), and then selecting a random sample of the remaining households of size needed to yield a total sample of 16 households per census block. The definition of a maternal death used was women 15 to 54 years of age who were pregnant at the time of death or who died within two months post-delivery.

The second main data source, the 2014 Village Potential Statistics (PODES) [16], is a census of villages that provides detailed information on the roughly 65,000 villages in Indonesia and the sub-districts and districts in which they are located. Three types of questionnaires were used: village-level, sub-district-level and district/city-level. Data were collected on population, environment, housing and settlements, educational facilities, social and cultural activities/institutions, recreation and entertainment, health facilities, nutrition and family planning, transportation and communication, land and its use, economy, security and information on village heads. Our main interest in the PODES data was information that described the supply environment for maternal health services.

### Operationalization of variables

The following variables were extracted from SUPAS for all ever-married women of reproductive age: age, parity, number of births in the previous five years, age and parity at time of all births in the previous five years, contraceptive use at the time of data collection, highest educational attainment, household economic status, and residence. We created a set of census block-level indicators that measured community-level maternal mortality risk and protective factors. These included the proportion of ever-married women of reproductive age who were using contraceptives; proportion of births at elevated risk due to too young or too old maternal age (i.e., under age 20 or over age 40); proportion of births at elevated risk due to high parity (i.e. parity four and above); proportion of women with primary-level education or lower, and proportion of women residing in households that were in the lowest two household wealth quintiles (classified as “very poor” and “poor”). Urban–rural location and island group (Java-Bali vs. others) were also included as variables to capture unmeasured differences in development and sociocultural factors. The operational definitions for all variables are provided in Table 1.

**Table 1:** Operational definitions of variables used in the analyses

Variable	Definition
Contraceptive prevalence rate	The proportion of women using a contraceptive method in a given census block = Number of ever married women using contraception divided by the number of ever married women
Contraceptive prevalence category	Coded 0 (Low) if contraceptive prevalence was less than 40%, 1 (Middle) if between 40 and 59%, 2 (High) if 60% or above
Proportion of high-risk births: maternal age	Coded 0 (Low) if proportion of women below age 20 or above age 40 in census block were less than 5% and 1 (High) otherwise
Proportion of high-risk births: parity	Coded 0 (low) if a census block had less than 5% of births to parity 4 or above, coded 1 (Middle) if a census block had ≥ 5% to 25% of births to parity 4 or above, and coded 2 (high) if a census block had > 25% of births to parity 4 or above
Proportion of low educated mothers	Low educated mother is defined as women of reproductive age having junior high school or less education. or less. Coded 0 (High educated) if a census block had less than 1% low-educated mothers; Coded 1 (Middle educated) if 1–40%; and Coded 2 (Low educated) if > 40%
Proportion of low socio-economic households	Household socio-economic status is divided into five quintile categories: (1) very poor, (2) poor, (3) middle, (4) rich, and (5) very rich. Low socio-economic household is defined a household in category ‘very poor’ or ‘poor’. Coded as 0 (Rich) if a census block had less than 1% low socio-economic households; Coded 1 (Middle) if the proportion of poor or very poor household was 1–49%; Coded 2 (Poor) if the proportion of very poor/poor households was 50% or more
District hospital population density	Number of hospitals per 1,000,000 district population. Coded 0 (Low) if the block census district hospital population density was less than 5 per 1,000,000 population; Coded 1 (Middle) if more than 15 per 1,000,000 population; and Coded 2 (High) if 5–15 per 1,000,000 population
Sub-district health center population density	Number of community health centers per 100,000 sub-district population. Coded 0 (Low) if the block census sub-district health center density was 5 or less per 100,000 population; Coded 1 (High) if more than 5 sub-district health centers per 100,000 population
Sub-district physician population density	Number of physicians per 100,000 sub-district population. Coded 0 (Low) if the block census sub-district physician density was 8 or less per 100,000 population; Coded 1 (High) if more than 8 physicians per 100,000 population
Village midwife population density	Number of midwives per 10,000 village population. Coded 0 (Low) if the block census village midwife density was 0.85 or less midwives per 10,000 population; Coded 1 (High) if more than 0.85 midwives per 10,000 population
Village TBA population density	Number of traditional birth attendants per 10,000 village population. Coded 0 (Low) if the block census village TBA density was less than 1 TBAs per 10,000 population; Coded 1 (Middle) if 1–4 TBAs per 10,000 population; Coded 2 (High) if more than 4 TBAs per 10,000 population
Island group	Coded 0 if the census block was located on the islands of Java or Bali; Coded 1 otherwise
Urban–rural	Coded 0 if the block census was located in an urban area; Coded 1 otherwise

Information on contraceptive use in SUPAS is limited to contraceptive status at the time of SUPAS data collection. This required the assumption that community contraceptive prevalence at the time of SUPAS data collection reflected census block differences in contraceptive practice during the five-year period prior to the SUPAS. We postulate that these were unlikely to have sufficiently dramatic to invalidate the assumption that community contraceptive prevalence measured in the 2015 SUPAS provides a valid proxy measure of relative levels of community contraceptive use during the 2010–2015 period.

PODES data were used to construct a series of variables describing the local supply environment for maternal health services in the form of population densities. These included the number of hospitals in the district in which sample census blocks were located per 1,000,000 population, the sub-district density of public health centers and physicians per 100,000 population, and the village density of midwives and traditional birth attendants (TBA) per 10,000 population. On the basis of these densities, we classified the access of respondents in a given census block to each type of health system asset as being high, medium or low. Further details may be found in Table 1.

### Statistical analyses

To measure the net impact of contraceptive use on maternal mortality, we estimated a series of log-linear regressions with census block MMRs in the five years preceding the 2015 SUPAS as the dependent or outcome variable. The unit of analysis in all regressions was census blocks (n = 40,728). The sampling weights calculated by the Indonesia Central Statistics Bureau (BPS), which corrected for unequal probabilities of selection of households, were applied to the data during analysis. Because of the skewness of the dependent variable and the large number of census blocks with no maternal deaths, we used a natural log transform with a small constant (one) added to MMR in each census block as the dependent variable in the analyses; that is,

Log-linear model:$Y^{\prime}_{{\text{i}}} = {\text{a}} + \sum {\text{bX}}_{{\text{i}}} + {\text{e}}_{{\text{i}}}$.

where:

$$Y^{\prime}_{{\text{i}}} = {\text{Ln }}\left[ {{1} + \left( {\left( {{\text{MMR}}_{{\text{i}}} } \right)*{1}00,000} \right)} \right]$$

$${\text{MMR}}_{{\text{i}}} = \left( {{\text{MD}}_{{\text{i}}} /{\text{LB}}_{{\text{i}}} } \right)*{1}00,000$$

MD_i_ = number of maternal deaths 2010–2015 in census block i, LB_i_ = number of live births 2010–2015 in census block i, X_i_ = vector of independent variables, α and β are regression coefficients to be estimated, and e_i_ = error term for census block i.

Visual inspection indicated that the distribution of the transformed dependent variable was improved, though not yet normalized. However, the distributional assumptions underlying the regressions become less of an issue with large sample sizes [17]. With large sample sizes, commonly used test statistics (e.g., p-values) rather quickly approach zero, and thus solely relying on p-values can lead to overstating the practical significance of empirical results [18]. Accordingly, we base our interpretation of results on effect sizes and their confidence intervals.

In the regression analyses, we first assess bivariable associations between the variables enumerated in Table 1 and maternal mortality, then a multivariable model with all variables included, followed by the identification of the most parsimonious model statistically. The latter was accomplished by backward elimination [19, 20]. In order to facilitate reader understanding of the results, we back-transform the log coefficients produced in the regressions for some of the key results in the Results and Discussion section of the article using the formula *e*^*β*^*−*1 (where β is the regression coefficient in log form). In addition to estimating the main effects of the variables considered in the analyses, we also estimated a regression model that included an interaction term to test the proposition that the effects of contraceptive use would be larger in districts where the maternal health infrastructure was less robust. To do this, we estimated the effects of contraceptive use within categories of the hospital population density variable, the latter being a proxy indicator for local health infrastructure.

## Results

A total of 227,990 live births and 1,593 maternal deaths were reported in the 2015 SUPAS as having occurred in the five years prior to the survey. Using the weighted SUPAS data, Ahmed [21] estimated a MMR for 2013–2015 of 237 per 100,000 live births (95% CI 201–274). The number of maternal deaths reported in census blocks ranged from zero to three, with no maternal deaths being reported in most (96.3%) census blocks. The distribution of maternal deaths by island group and time of death is shown in Table 2. Nationally, about 43% of reported maternal deaths occurred at the time of delivery, with roughly equal proportions occurring during pregnancy or postpartum. Only minor variations by island group are observed.

**Table 2:** Distribution of maternal deaths by island group and time of death

Island group	Time of death			Total
	Pregnancy	Delivery	Postpartum	
Eastern Indonesia	27.7	43.3	29.1	358
Sulawesi	27.9	43.9	28.3	244
Kalimantan	22.5	46.5	31.0	142
Sumatera	28.6	38.8	32.6	429
Java-Bali	28.6	38.8	32.6	420
Total	26.9	43.3	29.8	1,593

Table 3 displays the distribution of census blocks by the community-level risk and protective factors included in the study and the bivariable associations of these factors with the transformed community maternal mortality ratios. Not surprisingly in view of the large sample size, all variables were associated with maternal mortality at the p < 0.05 level or above, although the nature of the associations were not necessarily as anticipated. Higher community contraceptive prevalence was, as anticipated, protective against maternal mortality. Higher community of prevalence of births to women less than 20 or above 40 years of age, parity four-plus births, women with low education levels and households in the lower two household wealth quintiles were risk factors. Regarding the maternal health service supply environment, higher population densities of hospitals at the district level and physicians at the sub-district level were protective, while higher densities of health centers at the sub-district level and of midwives and traditional births attendants at the village level were associated with higher rather than lower maternal mortality at the bivariable level of analysis. Residence outside of the islands of Java and Bali and in rural areas was associated with elevated community maternal mortality levels. With regard to effect/association size, the strongest associations observed (i.e., coefficients of 0.15 or above) were (by order of magnitude) prevalence of low-wealth households, residence outside of Java-Bali, high density of TBAs, prevalence of high-parity births, prevalence of low-educated women and high density of hospitals.

**Table 3:** Bivariate associations between selected risk and protective factors and community maternal mortality ratios

Factors	N	Coef	95% CI	
Contraceptive prevalence rate (CPR)	40,728	− 0.142^*^	− 0.341	− 0.263
*Contraceptive use level category*				
Low (CPR < 40%)	9,465	0.000	−	−
Middle (CPR 40–59%)	12,254	− 0.041	− 0.091	0.011
High (CPR > 60%)	19,009	− 0.066^*^	− 0.112	− 0.019
*Proportion of high-risk births-maternal age*				
Low (< 5%)	24,445	0.000	–	−
High (≥ 5%)	16,283	0.086^*^	0.048	0.123
*Proportion of high-risk births-parity*				
Low (< 5%)	22,994	0.000	−	−
Middle (5–25%)	8,027	0.134^*^	0.086	0.182
High (> 25%)	9,707	0.239^*^	0.194	0.283
*Proportion of low educated mother (LEM)*				
High education (LEM < 1%)	16,847	0.000	–	–
Middle education (LEM 1–40%)	12,310	0.116^*^	0.072	0.159
Low education (LEM > 40%)	11,571	0.164^*^	0.119	0.208
*Proportion low socio-economic households (LSE)*				
Rich (LSE ≤ 1%)	5,846	0.000	–	–
Middle (LSE 1–50%)	20,376	0.128^*^	0.074	0.183
Poor (LSE > 50%)	14,506	0.346^*^	0.289	0.403
*District hospital density*				
Low (< 5 per 1,000,000 population)	10,390	0.000	–	–
Middle (5–15 per 1,000,000 population)	14,799	− 0.098^*^	− 0.146	− 0.052
High (> 15 per 1,000,000 population)	15,539	− 0.162^*^	− 0.208	− 0.115
*Sub-district health center density*				
Low (5 or less per 100,000 population)	17,244	0.000	–	–
High (> 5 per 100,000 population)	23,484	0.078^*^	0.041	0.116
*Sub-district health physician density*				
Low (8 or less per 100,000 population)	21,979	0.000	–	–
High (> 8 per 100,000 population)	18,749	− 0.057^*^	− 0.093	− 0.021
*Village midwife density*				
Low (0.85 or less per 10,000 population)	17,575	0.000	–	–
High (> 0.85 per 10,000 population)	23,153	0.052^*^	0.015	0.089
*Village TBA density*				
Low (< 1 per 10,000 population)	17,859	0.000	–	–
Middle (1–4 per 10,000 population)	12,524	0.061^*^	0.018	0.109
High (5 or more per 10,000 population)	10,345	0.226^*^	0.143	0.221
*Island Group*				
Java-Bali	16,005	0.000	–	–
Outside of Java-Bali	24,723	0.226^*^	0.189	0.264
*Urban–Rural*				
Urban area	19,169	0.000	–	–
Rural area	21,559	0.067^*^	0.031	0.104

*p < 0.05

Two sets of multivariable log-linear regression results are displayed in Table 4. In the first model, all variables included in the study were forced into the regression. In the second model, we parsed variables from the first model using a backward elimination process [19, 20], yielding the most parsimonious multivariate results. Only six of the 12 variables were retained in the final model—two related to contraceptive use/family planning, two related to the local supply environment for maternal health services (hospital and TBA population density), a socioeconomic indicator and a broad place of residence indicator (island group).

**Table 4:** Results of log-linear regressions of maternal mortality risk and protective factors on community maternal mortality ratios—full and reduced form models

Factors	Full model			Final model		
	Coef.	95% CI		Coef.	95% CI	
Contraceptive prevalence rate (CPR	− 0.064	− 0.147	0.081	− 0.073^*^	− 0.154	− 0.009
*Proportion of high-risk births—maternal age*						
Low (<5%)	0	–	–			
High (≥5%)	0.028	− 0.01	0.067			
*Proportion of high-risk births—parity*						
Low (<5%)	0	–	–	0	–	–
Middle (5–25%)	0.058^*^	0.009	0.109	0.076^*^	0.027	0.125
High (>25%)	0.125^*^	0.077	0.173	0.142^*^	0.094	0.189
*Proportion of low educated mother (LEM)*						
High education (LEM <1%)	0	–	–			
Middle education (LEM 1–40%)	0.028	− 0.018	0.074			
Low education (LEM >40%)	0.044	− 0.006	0.094			
*Proportion of low socio-economic households (LSE) *						
Rich (LSE ≤1%)	0	–	–	0	–	–
Middle (LSE 1–50%)	0.091^*^	0.032	0.151	0.077^*^	0.019	0.134
Poor (LSE >50%)	0.214^*^	0.142	0.287	0.193^*^	0.126	0.261
*District hospital density *						
Low (<5 per 1,000,000 population)	0	–	–	0	–	–
Middle (5–15 per 1,000,000 population)	− 0.032	− 0.079	0.016	− 0.031	− 0.078	0.018
High (>15 per 1,000,000 population)	0.059^*^	− 0.113	− 0.007	− 0.045^*^	− 0.096	− 0.006
*Sub-district health center density *						
Low (5 or less per 100,000 population)	0	–	–			
High (>5 per 100,000 population)	− 0.032	− 0.074	0.009			
*Sub-district physician density *						
Low (8 or less per 100,000 population)	0	–	–			
High (>8 per 100,000 population)	0.004	− 0.038	0.048			
*Village midwife density *						
Low (0.85 or less per 10,000 population)	0	–	–			
High (>0.85 per 10,000 population)	− 0.035	− 0.078	0.008			
*Village TBA density *						
Low (<1 per 10,000 population)	0	–	–	0	–	–
Middle (1–4 per 10,000 population)	0.035^*^	0.011	0.081	0.025	− 0.02	0.069
High (5+ per 10,000 population)	0.120^*^	0.067	0.173	0.103^*^	0.053	0.154
*Region*						
Java-Bali	0	–	–	0	–	–
Outside of Java-Bali	0.132^*^	0.086	0.177	0.113^*^	0.072	0.156
*Urban–rural*						
Urban area	0	–	–			
Rural area	− 0.052	− 0.099	0.005			

*p < 0.05

Our results indicate that residence on the islands of Java or Bali is associated with a 11.3% reduction in the community maternal mortality ratio net of all other factors considered in the analyses. Similarly, communities in which 50% of households fall into the lowest two national household wealth quintiles had community maternal mortality ratios that were nearly 20% higher than communities with no very poor or poor households, while communities with 1–49% of households falling into the lowest two household wealth quintiles had community maternal mortality ratios that were 7.7% higher.

Regarding the local supply environment for health services, communities with the lowest hospital densities had community maternal mortality ratios that were about 5% higher than communities with high hospital densities (p < 0.10). The opposite was the case for TBAs, where communities falling into the highest density category had 10% higher community maternal mortality ratios compared to communities falling into the lowest density category.

Of prime interest in the current research is the effects of the family planning-related variables. Our estimates indicate that for each percentage point increase in contraceptive prevalence, community maternal mortality ratios under the current level context were reduced by 7.0 points net of the other factors included in the final regression model (calculated as e^β^−1, where β is the regression coefficient in the final model in Table 4). The maternal parity risk variable, which reflects the impact of prior and current contraceptive use, also indicates a protective effect. The community maternal mortality ratio in communities in which 25% or more of births during the 2010–2015 period were parity four-plus births was 14.2% higher than in communities with zero parity four-plus births net of the effects of other variables considered.

As part of the analyses, we tested the proposition that contraceptive practice would have an even larger impact in settings in which the health service supply environment was less well developed. To test this proposition, we included contraception-supply environment interaction terms in the final regression from Table 4 that examined the effects of contraceptive use on community maternal mortality for different levels of district hospital population density. However, the interaction terms failed to achieve statistical significance and the effect sizes were small (data not shown), and thus we found no solid evidence to support the above proposition.

## Discussion

Prior studies using national and cross-national data have demonstrated that contraceptive use has been responsible for large-scale reductions in maternal mortality globally [4–9, 11]. Using two geographically detailed data sources from Indonesia, we sought to better understand the interplay of contraceptive use and other risk and protective factors for maternal mortality at the community level.

The study findings are consistent with prior literature in demonstrating a significant protective effect of contraceptive use [4–9, 11], in the present study net of the effects of a variety of other risk and protective factors. Our estimates indicate that in Indonesia during 2010–2015, for each percentage point increase in contraceptive prevalence community maternal mortality ratios were 7 points lower after adjustment for other risk and protective factors. Contraceptive use is also implicated in the findings concerning the maternal parity risk variable, which reflects the impact of prior contraceptive use as well as during the 2010–2015 reference period. The community maternal mortality ratios in communities in which 25% or more of births during the 2010–2015 period were parity four-plus births were 14.2% higher than in communities with zero parity four-plus births. The incremental health risks associated with higher parity pregnancies appear to be compounded in the Indonesian case by lower rates of health facility deliveries for higher-order births. The 2017 Indonesian Demographic and Health Survey (IDHS) indicates that only 63% of parity 4–5 births and 47% of parity 6-plus births in the five years prior to the survey were delivered at health facilities in comparison with 78% of parity one and 74% of parity 2–3 births [22].

Only one of the health service supply-side variables was found to be protective against maternal mortality (albeit with modest effect size)—higher hospital population density, a finding that is intuitively sensible given the importance of handling obstetric emergencies in preventing maternal deaths. There are several possible explanations for the failure to observe stronger supply-side effects. One is that the indicators used in the study capture the quantity but not the quality of health services available. A second possibility is that population density might not be a sufficiently sensitive indicator of physical access to health facilities and services as distances to such health assets could be much larger in settings with widely dispersed populations. Finally, it might be the case that existing health assets are not being fully taken advantage of due to preferences for more traditional service providers such as TBAs, with TBAs being a marker of existence traditional practice that hampers care seeking with more skilled service providers. An earlier study in West Java Province found that physical distance and financial limitations were the major constraints limiting greater use of trained attendants and institutional deliveries [23]. Unfortunately, we are unable to empirically assess the relative merits of the alternative explanations based upon the available data.

That the population density of TBAs at the village level emerged as a risk factor with moderate effect size merits attention. Globally, improving access to skilled health personnel for childbirth has been a priority for improving maternal health for many years [24, 25]. Updated WHO recommendations [26] support the use of lay health workers, including trained TBAs, to promote the uptake of several maternal and newborn-related health care behaviors working collaboratively with skilled birth attendants and not as substitutes for more highly trained personnel. Our findings suggest that additional TBA capacity building and clearer role definitions are needed if TBAs are to contribute to reducing maternal mortality in Indonesia.

Residence on islands other than Java and Bali was associated with 11% higher community maternal mortality ratios after adjustment for the other factors considered in the analyses. Such differences in health outcomes are generally thought to reflect geographic inequities in health resources, with provinces and districts in the eastern part of the country being relatively under-developed compared to Java-Bali [27]. Our findings suggest that factors other than the population density of health facilities and service providers are at play. Plausible explanations include systematic variations in the quality of and in demand for “modern” maternal health services.

Regarding socioeconomic inequities, communities in which 50% or more of households were classified as poor or very poor had community maternal mortality rates that were nearly 20% higher than communities with no very poor or poor households. This result implicates financial barriers to the use of maternal health services in the form of service fees, transport and opportunity costs. The importance of financial barriers is also suggested in the 2017 IDHS finding that only 45% of deliveries in the five years preceding the survey to women in the lowest wealth quintile were institutional deliveries vs. 94% to women in the highest wealth quintile [17]. This is important as poorer women have a higher incidence of iron deficiency anemia and are at greater risk of hypertensive disease in pregnancy among other deficiencies associated to higher risk of maternal mortality [2]. Low household wealth does not, however, appear to a serious a barrier to contraceptive use as women in the lowest wealth quintile households had contraceptive prevalence rates in the 2017 IDHS data that were comparable to those in the top two household wealth quintiles [17]. The new national social health insurance scheme, the *Jaminan Kesehatan Nasiona*l (JKN), which was in its second year at the time of the 2015 SUPAS, should help alleviate these inequities, but will require significant further investment in order to reach universal coverage and be able to provide sufficient quality services to an expanded consumer base.

Several limitations of the study should be noted. First, because the information collected on maternal deaths in the SUPAS data was limited to place of death; age at the time of death; and whether the death occurred during pregnancy, during delivery or post-delivery, we lack information on risk and protective factors preceding reported deaths as would be required in order to undertake analyses with individual women as the unit of analysis. We addressed this issue by undertaking an undertook an ecological analysis in which we related maternal mortality risk and protective factors measured at the community level to the ratio of maternal deaths to live births in each census block during the 5 years 2010–2015.

Second, Information on contraceptive use in SUPAS is limited to contraceptive status at the time of SUPAS data collection. This required the assumption that community contraceptive prevalence at the time of SUPAS data collection reflected census block differences in contraceptive practice during the 5-year period prior to the SUPAS. At the national level, contraceptive prevalence was stagnant during this reference period (61% in 2007 and 64% in 2017 [22]. While there is certain to have been some variability in the rate of change in contraceptive prevalence across subnational geographic units, we postulate that these were unlikely to have sufficiently dramatic to invalidate the assumption that community contraceptive prevalence measured in the 2015 SUPAS provides a valid proxy measure of relative levels of community contraceptive use during the 2010–2015 period.

Finally, because the unit of analysis in the study was the census block, the statistical relationships measured in the study are ecological or group relationships from which it is not valid to infer that the relationships will hold at the individual level of analysis. In order to avoid the “ecological fallacy,” inferences must be limited to group or community characteristics and aggregate behaviors.

## Conclusions

Community contraceptive prevalence made a significant contribution to reducing maternal mortality in Indonesia net of other maternal mortality risk and protective factors during the 2010–2015 period. While further increases in contraceptive use will be welcome, increased health system responsiveness to the needs of pregnant women and reductions in socioeconomic and geographic disparities in maternal health services will be needed if Indonesia is to reach the 2030 SDG maternal mortality goal.

## Data Availability

Requests for access to the data analyzed in this study should be directed to the Indonesian Badan Pusat Statistik (Central Statistics Bureau)—website https://www.bps.go.id.

## Abbreviations

BKKBN: National Population and Family Planning Board
GoI: Government of Indonesia
GNI: Gross national income
JKN: Jaminan Kesehatan Nasional
MoH: Ministry of Health
MMR: Maternal mortality ratio
PODES: Village potential survey
SDG: Sustainable development goals
SUPAS: Intercensal population survey
TBA: Traditional birth attendant
WHO: World Health Organization
